# Supplementary material for: Liposomes Loaded with Everolimus and Coated with Hyaluronic Acid: A Promising Approach for Lung Fibrosis
Source: Int J Mol Sci. 2021 Jul 20;22(14):7743. doi: 10.3390/ijms22147743 (PMC8303794; doi:10.3390/ijms22147743)
Supplement: Supplementary file 1 [file ijms-22-07743-s001.zip › ijms-1264679-supplementary.pdf]

Supplementary information

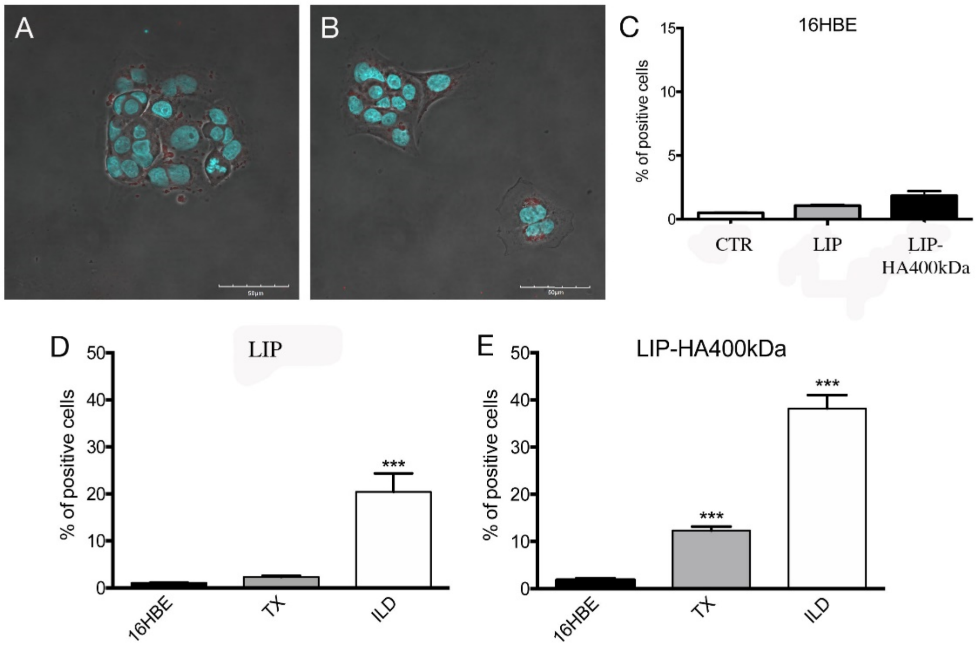

Figure S1. Internalization analysis and quantification of LIP and LIP-HA400kDa in CD44-negative cells (16HBE). (A and B) Confocal images of 16HBE treated with (A) LIP and (B) LIP-HA400kDa after 4 h. Nuclei = DAPI; liposomes = red. (C) Flow cytometry analysis of 16HBE incubated with LIP and LIP-HA400kDa. Data are represented as mean of percentage of positive cells ± SD. (D and E) Comparison of flow cytometry quantification of (D) LIP and (E) LIP-HA400kDa internalized by 16HBE, TX and CTD-ILD. Data are represented as mean of percentage of positive cells ± SD. ***, $p < 0.01$ vs. CTR.

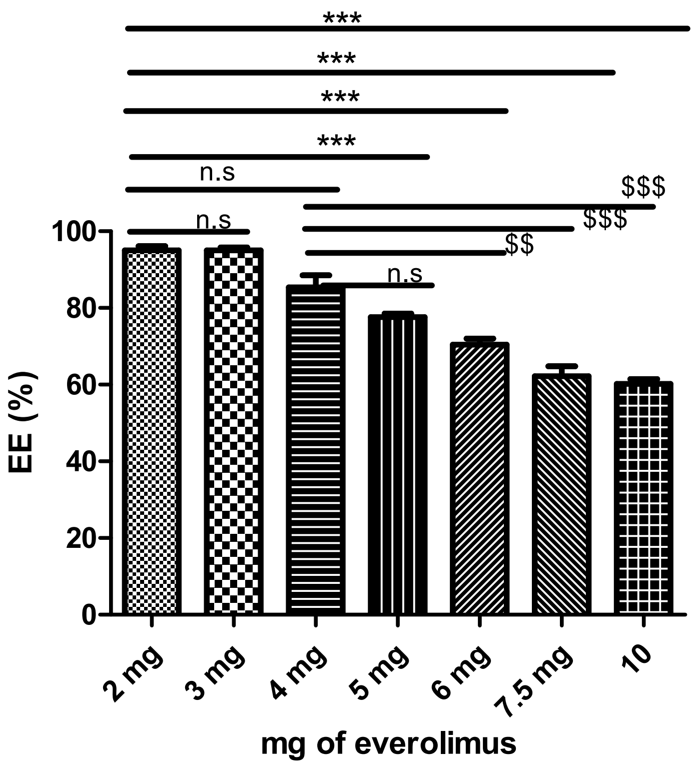

Figure S2. EE of liposomes as a function of mg of everolimus added in the PEGylated liposome formulation. ***, $p < 0.001$ vs. 2 mg, $$, $p < 0.01$ vs. 4 mg, $$$, $p < 0.001$ vs. 4 mg. (One-way ANOVA followed by Tukey post-hoc).

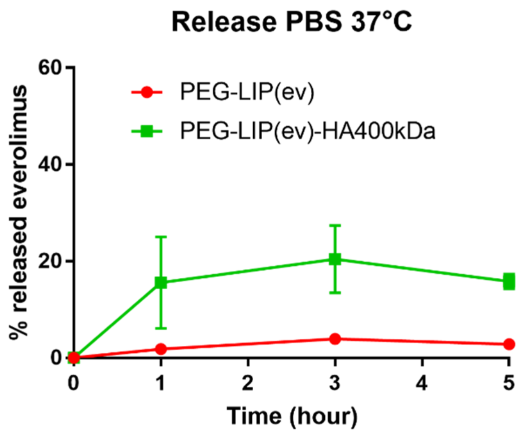

Figure S3. Everolimus release profile assessed at 37 °C in PBS at time points of 0, 1, 3 and 5 h.
